# Associations of thigh muscle fat infiltration with isometric strength measurements based on chemical shift encoding-based water-fat magnetic resonance imaging

**DOI:** 10.1186/s41747-019-0123-4

**Published:** 2019-11-20

**Authors:** Stephanie Inhuber, Nico Sollmann, Sarah Schlaeger, Michael Dieckmeyer, Egon Burian, Caroline Kohlmeyer, Dimitrios C. Karampinos, Jan S. Kirschke, Thomas Baum, Florian Kreuzpointner, Ansgar Schwirtz

**Affiliations:** 1 Department of Sport and Health Sciences, Technische Universität München, Georg-Brauchle-Ring 60/62, 80992 Munich, Germany; 2 Department of Diagnostic and Interventional Neuroradiology, Klinikum rechts der Isar, Technische Universität München, Ismaninger Str. 22, 81675 Munich, Germany; 3 Department of Diagnostic and Interventional Radiology, Klinikum rechts der Isar, Technische Universität München, Ismaninger Str. 22, 81675 Munich, Germany

**Keywords:** Healthy volunteers, Magnetic resonance imaging, Muscle contraction (isometric), Muscle strength, Thigh

## Abstract

**Background:**

Assessment of the thigh muscle fat composition using magnetic resonance imaging (MRI) can provide surrogate markers in subjects suffering from various musculoskeletal disorders including knee osteoarthritis or neuromuscular diseases. However, little is known about the relationship with muscle strength. Therefore, we investigated the associations of thigh muscle fat with isometric strength measurements.

**Methods:**

Twenty healthy subjects (10 females; median age 27 years, range 22–41 years) underwent chemical shift encoding-based water-fat MRI, followed by bilateral extraction of the proton density fat fraction (PDFF) and calculation of relative cross-sectional area (relCSA) of quadriceps and ischiocrural muscles. Relative maximum voluntary isometric contraction (relMVIC) in knee extension and flexion was measured with a rotational dynamometer. Correlations between PDFF, relCSA, and relMVIC were evaluated, and multivariate regression was applied to identify significant predictors of muscle strength.

**Results:**

Significant correlations between the PDFF and relMVIC were observed for quadriceps and ischiocrural muscles bilaterally (*p* = 0.001 to 0.049). PDFF, but not relCSA, was a statistically significant (*p* = 0.001 to 0.049) predictor of relMVIC in multivariate regression models, except for left-sided relMVIC in extension. In this case, PDFF (*p* = 0.005) and relCSA (*p* = 0.015) of quadriceps muscles significantly contributed to the statistical model with *R*^2^_adj_ = 0.548.

**Conclusion:**

Chemical shift encoding-based water-fat MRI could detect changes in muscle composition by quantifying muscular fat that correlates well with both extensor and flexor relMVIC of the thigh. Our results help to initiate early, individualised treatments to maintain or improve muscle function in subjects who do not or not yet show pathological fatty muscle infiltration.

## Key points

- Magnetic resonance imaging detects changes in muscle composition by quantifying muscular fat.
- Muscular fat correlates well with extensor and flexor strength at the thigh.
- Muscular fat, not cross-sectional area, can predict muscle strength in thigh muscles.
- The interaction between muscular fat and strength could become the basis for a biomarker for muscle quality and function.

## Background

Previous research has demonstrated alterations in fat composition of thigh muscles due to various pathological conditions primarily when they are already in a chronic stage, including musculoskeletal disorders, metabolic diseases, and neuromuscular diseases (NMD) [1–9]. Furthermore, physical exercise and training have shown to entail measurable changes in the thigh musculature [10–12]. Such alterations can nowadays be captured non-invasively by means of imaging, with magnetic resonance imaging (MRI) being at the forefront particularly thanks to the ability to perform qualitative and quantitative assessments of the human body fat composition *in vivo* [13].

For the purpose of assessing muscle fat composition, anatomical T1- and T2-weighted MRI is conventionally applied [13]. Based on anatomical imaging, the cross-sectional area (CSA) of muscles can be calculated, which can be used as a structural measure of muscle hypertrophy or atrophy [14–16]. More advanced MRI-based methods including magnetic resonance spectroscopy and chemical shift encoding-based water-fat imaging enable the extraction of parameters like the proton density fat fraction (PDFF) [13, 17]. In contrast to conventional T1- and T2-weighted MRI, chemical shift encoding-based water-fat MRI allows for robust quantitative and, thus, more objective evaluation of muscle composition whilst simultaneously enabling anatomical assessment [18–20]. In this context, thigh muscles represent a region of high interest for MRI investigation thanks to good magnetic field homogeneity, minimum motion artefacts, and the knowledge about disease-characteristic features of some pathologies in these muscles. Additionally, it is possible to perform precise strength measurements at thigh muscles, thus allowing for direct links to changes in quantitative MRI to provide insights into muscle quality and (dys-)function [21].

However, evidence of association between thigh muscle fat composition and muscle strength is scarce [22–25]. Inverse relationships were reported for the PDFF and strength at the thigh in patients with NMD [1, 8]. However, whether the PDFF of extensor and flexor thigh muscles improves the prediction of thigh isometric strength in extension and flexion beyond the CSA is not clear. To confirm a close relationship between thigh muscle PDFF and strength is clinically important. The thigh muscles are one of the largest muscle groups of the human body and thus important target muscles to diagnose and monitor different diseases affecting local and whole-body muscle pathologies such as knee osteoarthritis, NMD, sarcopenia, and tumour cachexia. Detecting and understanding changes in thigh muscle quality, which are based on muscle fat compositions that correlate with muscle strength, could support initiating early and individualised treatment protocols to maintain or improve muscle function prior to clinical diagnosis. Therefore, we investigated the association of thigh muscle fat with isometric strength measurement using chemical shift encoding-based water-fat MRI and a rotational dynamometer in healthy volunteers. The hypothesis is that thigh muscle PDFF improves the prediction of muscle strength measurements beyond muscle CSA.

## Methods

### Subjects

Twenty healthy volunteers (10 females; median age 27 years, range 22–41 years) were recruited. Age between 20 and 45 years and body mass index (BMI) between 23 and 33 kg/m^2^ were defined as inclusion criteria to reflect the average population with a rather broad BMI range (median BMI of the study population 26.7 kg/m^2^, range 22.2–31.8 kg/m^2^). Completing the international physical activity questionnaire in short-form ensured that all subjects had a moderate level of physical activity (referring to the scoring protocol: 600–1500 metabolic equivalent of task-min/week) [26, 27]. Exclusion criteria were (1) any history of high-performance sports, (2) any history of metabolic diseases, NMD, previous knee or thigh muscle injuries, (3) general contraindications for MRI (*e.g.*, cochlear implants), and (4) implanted foreign bodies at the level of the upper leg.

MRI and strength measurements were scheduled within 5 days to avoid any mismatch between the measured strength and fat parameters as obtained by MRI-related analyses. In case that both parts of the measurement took place on the same day, MRI was carried out before strength measurements with the intent to not capture potential modifications in muscular structures due to strength measurement.

The study was approved by the local institutional review board and conducted in accordance with the Declaration of Helsinki. All subjects gave written informed consent on MRI examinations, isometric strength measurements, and publication of identifying images prior to participation in the study. Data are accessible upon request.

### MRI

Subjects underwent 3-T MRI of the bilateral thigh muscles (Ingenia, Philips Healthcare, Best, The Netherlands). Scanning was performed in supine position using a built-in-the-table 12-channel posterior coil and a 16-channel anterior coil.

An axially-prescribed, six-echo three-dimensional spoiled gradient-echo sequence was acquired for chemical shift encoding-based water-fat separation with the following parameters: repetition time (TR)/echo time (TE) min/ΔTE = 6.4/1.1/0.8 ms; field of view 220 × 401 × 252 mm^3^; acquisition matrix 68 × 150, voxel size 3.2 × 2.0 × 4.0 mm^3^; frequency encoding direction left-to-right L/R, no sensitivity encoding. Scan time was 1 min and 25 s per stack. The six echoes were acquired in a single TR using non-flyback (bipolar) read-out gradients with two axial stacks being obtained consecutively to cover the entire thigh region from the hip down to the superior edge of the patella. A flip angle of 3° was used to minimise T1 bias effects [18, 28].

### Imaging-based fat quantification

The imaging data were first processed by applying a phase error correction and a complex-based water-fat decomposition considering a pre-calibrated seven-peak fat spectrum and a single T2* [29, 30]. We used the multi-echo mDIXON fat quantification routine of the vendor. The PDFF maps were computed as the ratio of the fat signal over the sum of fat and water signals. The axial PDFF maps of both stacks of each subject were stored for segmentation.

### Segmentation

**Fig. 1:**
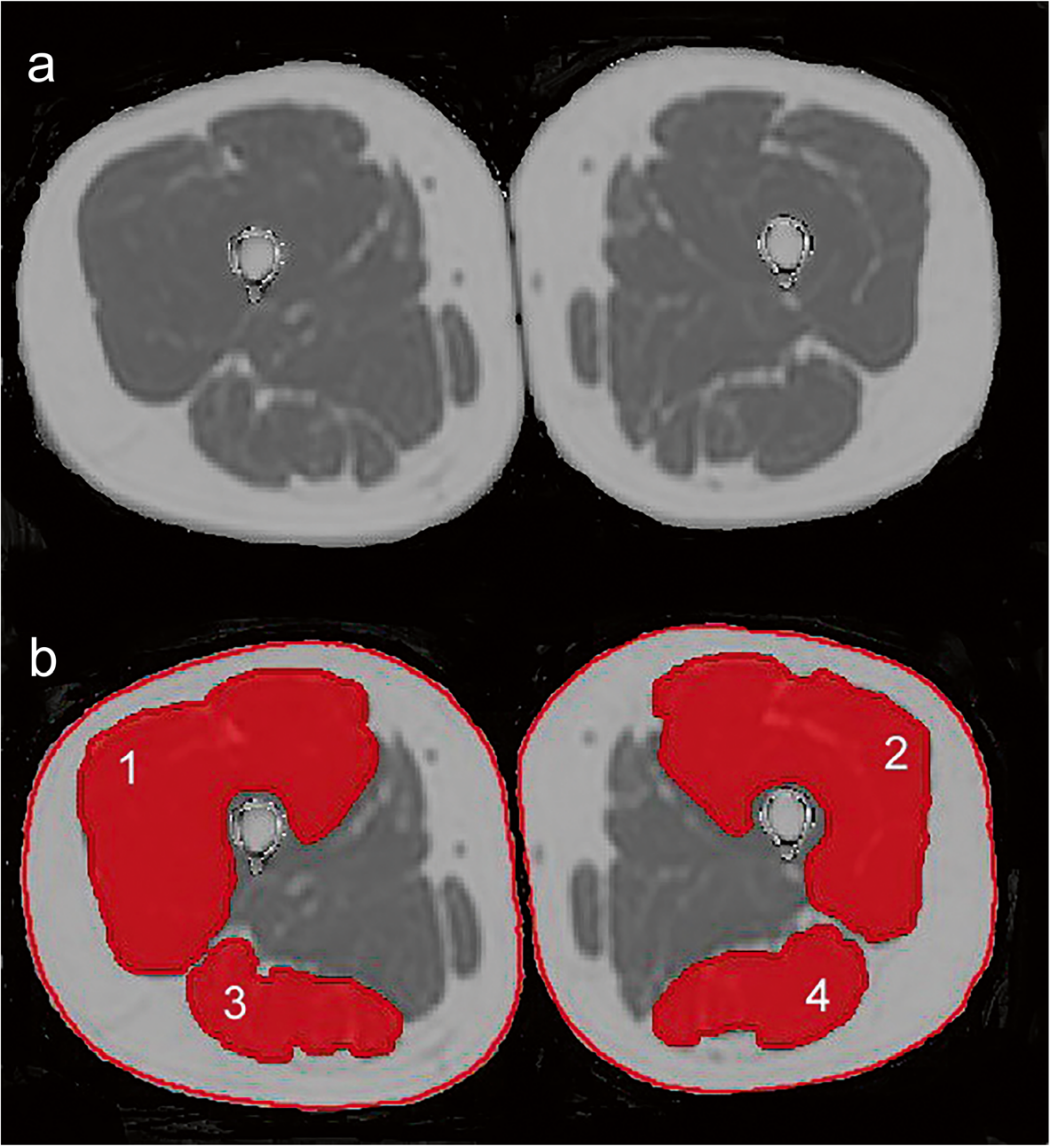
Chemical shift encoding-based water-fat magnetic resonance imaging (MRI) and placement of regions of interest (ROIs). **a** Representative proton density fat fraction (PDFF) map. **b** PDFF map with superimposition of manually segmented muscle compartments defined as ROIs: (1) right quadriceps muscle, (2) left quadriceps muscle, (3) right ischiocrural muscles, and (4) left ischiocrural muscles. The red lines around the thigh represent the segmentation of the entire thigh contour

Segmentations of the quadriceps femoris muscles and ischiocrural muscles of both sides were performed within the ten most central slices depicting the thigh muscles (Fig. 1). Segmentations were done on the PDFF maps using MITK (http://mitk.org/wiki/The_Medical_Imaging_Interaction_Toolkit_(MITK); German Cancer Research Center, Division of Medical and Biological Informatics, Medical Imaging Interaction Toolkit, Heidelberg, Germany). The thigh muscles were manually segmented on the first, fifth, and tenth slice. Polygonal regions of interest (ROIs) were carefully placed in these axial slices. We then used the two-dimensional interpolation tool of MITK to obtain segmentations of the remaining axial slices. These segmentations were manually corrected. The ROIs were placed at the outer muscle contour whilst carefully avoiding the inclusion of subcutaneous fat or muscle fat interfaces [2, 22]. Subsequent to ROI placements, the CSA (in mm^2^) and PDFF (in %) of the quadriceps and ischiocrural muscles were extracted separately for both sides by averaging the respective values obtained from the ten consecutive slices per side. Additionally, the entire thigh contour was segmented in the same ten slices (Fig. 1), followed by calculation of the mean thigh CSA per subject. A relative CSA (relCSA, in %) of quadriceps and ischiocrural muscles was determined by dividing the respective muscle CSA by the entire thigh CSA. All segmentations were performed by a radiologist with 8 years of experience in musculoskeletal imaging. Good reproducibility of measurements following this approach was previously reported [22].

### Isometric muscle strength measurements

**Fig. 2:**
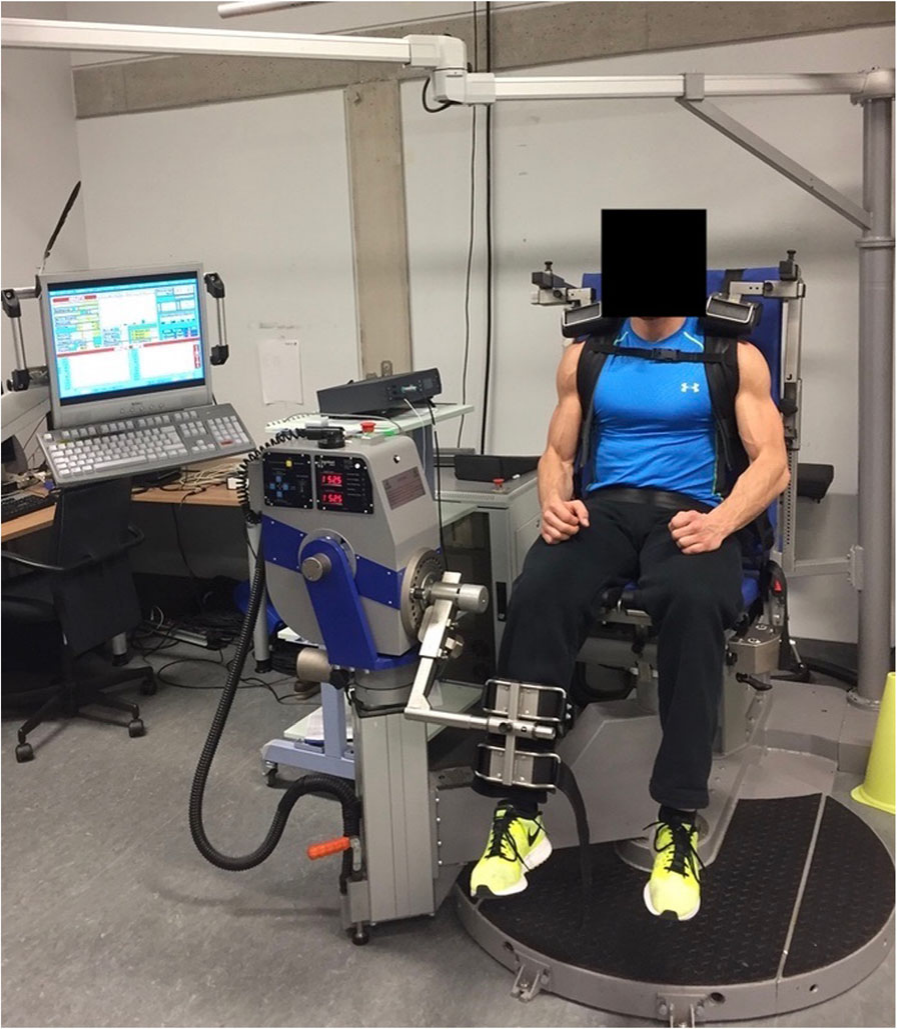
Setup for measurements of the maximum voluntary isometric contraction (MVIC) with a rotational dynamometer

The maximum voluntary isometric contraction (MVIC) in single-joint knee extension and flexion was measured at the thigh bilaterally with a rotational dynamometer (IsoMed 2000, D&R Ferstl GmbH, Hemau, Germany). Substantiated by measuring the isometric peak torque in Newton per metre (in N*m), the MVIC was produced in knee extension at 60° and knee flexion at 35°, which are the joint angles with the ideal muscular strength-length relationship to anticipate the real MVIC [31–34]. At the beginning of the measuring visit, the isokinetic rotational dynamometer was calibrated exactly on subjects’ individual body dimension. Subjects were seated in an upright sitting position and were fixed by hip and shoulder belts as well as shoulder pads, and two adjustable straps were used to fix the leg at the pad of the lever arm in the correct measuring position (Fig. 2).

Following the individual calibrating, the subjects had to perform a standardised warm-up exercise. After 10 min on a cycling ergometer at 70–80 rpm (revolutions per minute) to activate the cardiovascular system, the volunteers were seated in the rotational dynamometer where ten dynamic repetitions with moderate intensity were performed. In the fixed measuring position, the subjects’ task was to extend or to flex the knee against the measuring pad on the back or front side of the lower leg with the individual maximum contraction of quadriceps or ischiocrural muscles. MVIC of each direction of movement (extension/flexion) was collected three times with 3 min of recovery in between, and the best value of muscle flexion and extension maximum isometric torque (in N*m) was respectively taken for data analysis [2, 22]. The choice of the starting leg and the direction of movement were randomised.

### Strength data collection method

The force transducer is located in the inside of the rotational lever arm of the used rotational dynamometer to transfer the measured values of maximum isometric torque to the software (proEMG, Prophysics AG, Switzerland). The value the tested leg produces whilst sitting in a resting position caused by the gravity was measured in each testing step. The measured absolute extension and flexion MVICs (in N*m) were adjusted for the individual BMI to obtain a relative MVIC (relMVIC, in N*m^3^/kg) for the left and right thigh muscles.

### Reproducibility of muscle strength measurements

All participants performed at least one initial training session. The visit’s aim was to become familiar with the measuring procedure and to train to afford and activate the maximum isometric strength. To ensure the reproducibility of strength measurements, each subject performed at least five to eight repetitions with a MVIC and 3 min of rest in between. A verifiable increase and decrease of the measured values was assured as the decisive factor for a potentially needed second training visit or the testing appointment. There were at least 3 days of rest between the visits and subjects were instructed to come to the measurements totally recovered (no physical activity the 2 days before visits).

### Statistical analyses

SPSS (version 20.0; IBM SPSS Statistics for Windows, Armonk, NY, USA) was used for all statistical analyses and generation of graphs. The level of statistical significance was set at *p* <  0.05 (two-sided). The Kolmogorov-Smirnov test indicated normal distribution of BMI, PDFF, relCSA, and relMVIC values, but not of age.

First, mean ± standard deviation was calculated for PDFF, relCSA, and relMVIC, separately for male and female subjects as well as for the right and left thigh muscles. Comparisons of these measures between genders were performed by using unpaired *t*-tests. Furthermore, Pearson correlation coefficients (*r*) were calculated between PDFF, relCSA, and relMVIC. Using these parameters, multivariate regression models were calculated to identify significant predictors of thigh muscle strength. Parameters were included in the regression models for *p* <  0.05.

## Results

### Proton density fat fraction and cross-sectional area measurements

Mean muscle PDFF was lower in males than females in the left and right quadriceps muscles (2.7 ± 1.3% *versus* 3.6 ± 1.3%, *p* = 0.162; 1.7 ± 1.3% *versus* 2.7 ± 1.3%, *p* = 0.105) and ischiocrural muscles (3.2 ± 1.6% *versus* 4.6 ± 2.0%, *p* = 0.091; 2.6 ± 1.9% *versus* 4.9 ± 2.4%, *p* = 0.025; Table 1). There were no significant differences in age or BMI between males and females (*p* = 0.280 and 0.684, respectively).

**Table 1:** Proton density fat fraction (PDFF), relative cross-sectional area (relCSA), and relative maximum voluntary isometric contraction (relMVIC) in extension and flexion

		Males	Females	*p* value
PDFF and relCSA				
PDFF quadriceps (%)	Left	2.7 ± 1.3	3.6 ± 1.3	0.162
	Right	1.7 ± 1.3	2.7 ± 1.3	0.105
PDFF ischiocrural (%)	Left	3.2 ± 1.6	4.6 ± 2.0	0.091
	Right	2.6 ± 1.9	4.9 ± 2.4	0.025
relCSA quadriceps (%)	Left	26.5 ± 4.0	21.3 ± 4.1	0.010
	Right	25.8 ± 4.2	21.8 ± 3.7	0.035
relCSA ischiocrural (%)	Left	8.3 ± 2.4	6.0 ± 2.4	0.045
	Right	7.5 ± 3.0	6.6 ± 1.6	0.418
RelMVIC in extension and flexion				
relMVIC in extension (N*m^3^/kg)	Left	8.0 ± 1.2	5.9 ± 1.0	< 0.001
	Right	8.8 ± 1.3	6.4 ± 0.9	< 0.001
relMVIC in flexion (N*m^3^/kg)	Left	4.0 ± 0.6	3.0 ± 0.5	< 0.001
	Right	4.3 ± 0.6	2.7 ± 0.7	< 0.001

Data are expressed as mean ± standard deviation

Males showed greater relCSA than females in the quadriceps muscles (26.5 ± 4.0% *versus* 21.3 ± 4.1%, *p* = 0.010; 25.8 ± 4.2% *versus* 21.8 ± 3.7%, *p* = 0.035) and ischiocrural muscles at both sides (8.3 ± 2.4% *versus* 6.0 ± 2.4%, *p* = 0.045; 7.5 ± 3.0% *versus* 6.6 ± 1.6%, *p* = 0.418; Table 1). Quadriceps muscles had a bigger relCSA than ischiocrural muscles for males and females (Table 1).

### Muscle strength measurements

RelMVIC in extension was higher in males compared to females in the left (8.0 ± 1.2 N*m^3^/kg *versus* 5.9 ± 1.0 N*m^3^/kg, *p* <  0.001) and right quadriceps muscles (8.8 ± 1.3 N*m^3^/kg *versus* 6.4 ± 0.9 N*m^3^/kg, *p* <  0.001; Table 1). Similarly, males had greater relMVIC in flexion than females in the left (4.0 ± 0.6 N*m^3^/kg *versus* 3.0 ± 0.5 N*m^3^/kg, *p* <  0.001) and right ischiocrural muscles (4.3 ± 0.6 N*m^3^/kg *versus* 2.7 ± 0.7 N*m^3^/kg, *p* <  0.001; Table 1). Quadriceps muscles showed approximately twice as much relMVIC than ischiocrural muscles at both thighs in males (mean quadriceps 8.4 ± 1.2 N*m^3^/kg *versus* mean ischiocrural 4.1 ± 0.6 N*m^3^/kg) and females (mean quadriceps 6.2 ± 1.0 N*m^3^/kg *versus* mean ischiocrural 2.9 ± 0.6 N*m^3^/kg; Table 1).

### Correlations and multivariate regression models

**Fig. 3:**
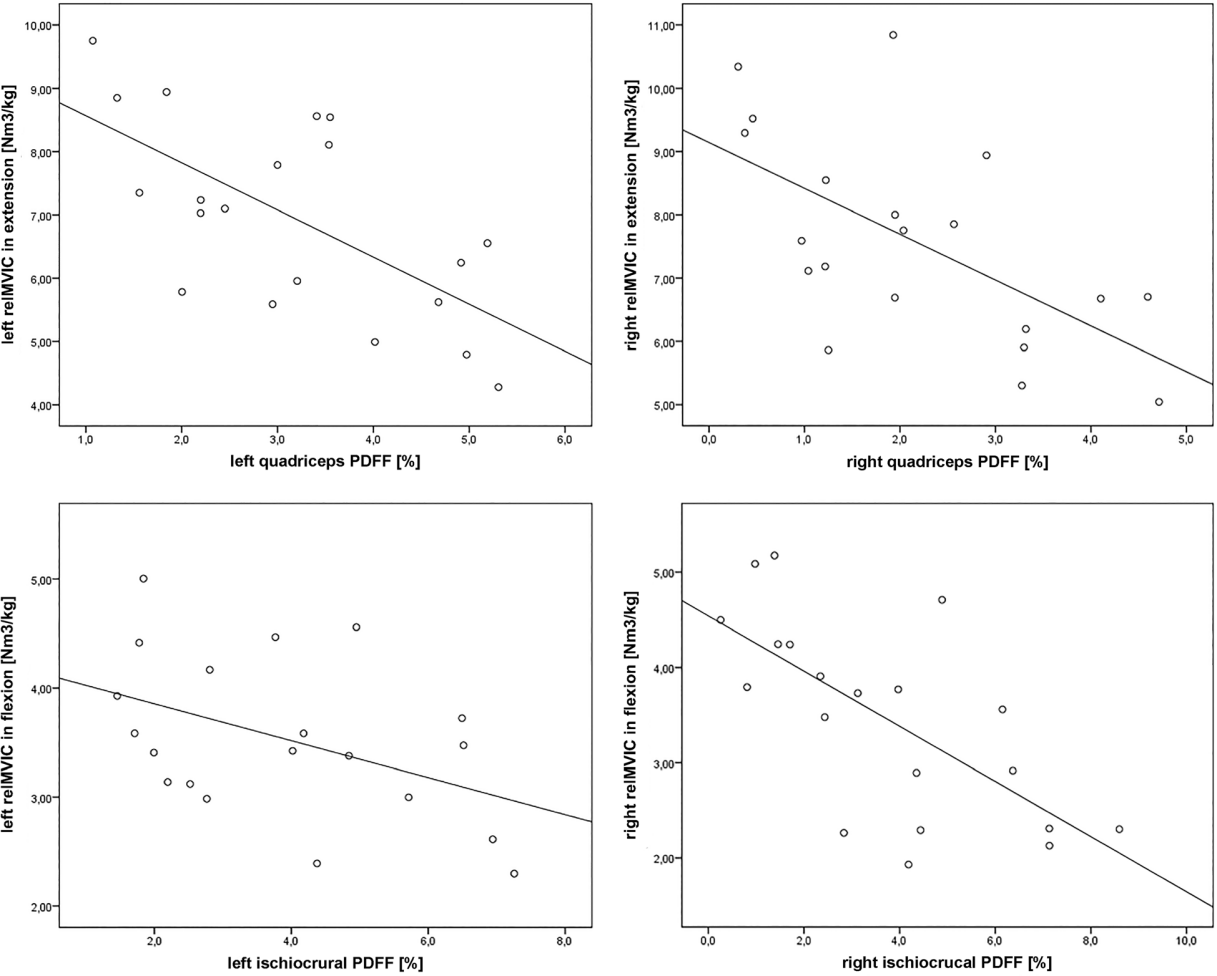
Correlation between the proton density fat fraction (PDFF) and relative maximum voluntary isometric contraction (relMVIC). Plots showing the association between the left or right relMVIC in extension or flexion (in N*m^3^/kg) and the PDFF (in %) of the left or right quadriceps or ischiocrural muscles

Significant correlations were revealed between the relMVIC in extension and the PDFF of the left (*r* = -0.649, *p* = 0.002) and right quadriceps muscle (*r* = -0.612, *p* = 0.004; Table 2, Fig. 3). RelMVIC in flexion was correlated significantly with the PDFF of the left (*r* = -0.446, *p* = 0.049) and right ischiocrural muscles (*r* = -0.676, *p* = 0.001; Table 2, Fig. 3).

**Table 2:** Correlation between the proton density fat fraction (PDFF) or relative cross-sectional area (relCSA) and relative maximum voluntary isometric contraction (relMVIC) in extension and flexion (males and females together)

			relMVIC		relCSA	
			Left	Right	Left	Right
Extension	PDFF	*r* *p* value	-0.649 0.002	-0.612 0.004	-0.284 0.225	-0.308 0.186
	relCSA	*r* *p* value	0.585 0.007	0.448 0.048	–	–
Flexion	PDFF	*r* *p* value	-0.446 0.049	-0.676 0.001	-0.125 0.599	-0.173 0.465
	relCSA	*r* *p* value	0.238 0.312	0.207 0.380	–	–

**Fig. 4:**
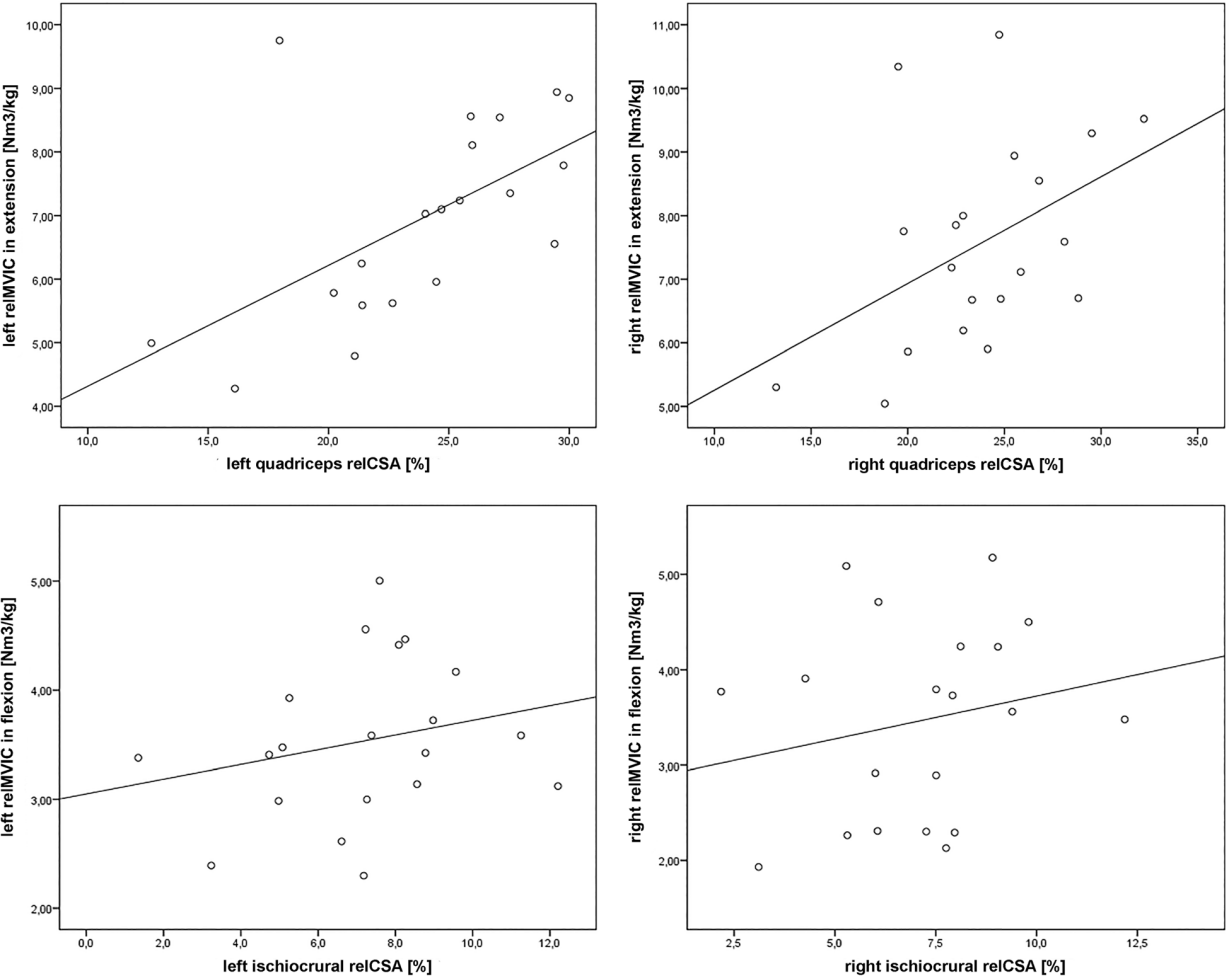
Correlation between the relative cross-sectional area (relCSA) and relative maximum voluntary isometric contraction (relMVIC). Plots showing the association between the left or right relMVIC in extension or flexion (in N*m^3^/kg) and the relative CSA (in %) of the left or right quadriceps or ischiocrural muscles

Furthermore, the relMVIC in extension was significantly associated with the relCSA of the left (*r* = 0.585, *p* = 0.007) and right quadriceps muscle (*r* = 0.448, *p* = 0.048; Table 2, Fig. 4). There was no significant correlation found between the relMVIC in flexion and relCSA of the ischiocrural muscles for the left and right sides (Table 2, Fig. 4).

PDFF, but not relCSA, was a statistically significant (*p* = 0.001 to 0.049) predictor of relMVIC in multivariate regression models, except for left-sided relMVIC in extension. In this case, PDFF (*p* = 0.005) and relCSA (*p* = 0.015) of the quadriceps muscles contributed significantly to the statistical model with *R*^2^_adj_ = 0.548.

## Discussion

This study used chemical shift encoding-based water-fat MRI at the thigh in healthy volunteers to extract the PDFF and relCSA of quadriceps and ischiocrural muscles bilaterally and a rotational dynamometer to assess relMVIC in extension and flexion. Muscle PDFF was a better predictor of the relMVIC than the relCSA. We observed significant differences in the relCSA of ischiocrural and quadriceps muscles, but also in the PDFF of ischiocrural muscles between males and females.

Grimm et al. [3] recently reported averaged PDFF values between 5.6 and 6.9% in healthy young males; however, in their investigation, PDFF measurements were derived from all thigh muscles and without distinct exclusion of intermuscular tissue. Specifically for the quadriceps muscles, a previous study [22] reported a mean intramuscular PDFF of 4.02% in a cohort of healthy males. Schlaeger et al. [2] performed the segmentation of individual thigh muscles as part of the MyoSegmenTUM_thigh database, with average PDFF values of 3.71% and 3.93% for the right and left quadriceps muscle as well as 4.38% and 4.44% for the right and left ischiocrural muscles by also focusing on healthy males and females. However, a distinction between genders has not been achieved in their study because only three healthy females were included in total [2]. Thus, the PDFF values obtained in the present study seem to be principally in the range of previously reported values.

Alterations in fat composition of thigh muscles have been shown in the context of diseases such as musculoskeletal disorders, metabolic diseases, and NMD [1–9]. Specifically, patients suffering from different types of muscular dystrophy, Pompe disease, sarcopenia, osteoarthritis, or type 2 diabetes mellitus showed increased PDFF or intramuscular fat when compared to healthy controls [1–9]. Although it has been hypothesised that increased fatty infiltration or transformation of thigh muscles is related to decrease in muscle strength [21, 23], studies correlating parameters such as the PDFF or absolute or relative CSA of thigh muscles with objective measurements of muscle strength are rare. An inverse relationship was revealed between the PDFF and strength at the thigh in patients with NMD [1, 8]. In these studies, the obtained strength measurements were acquired with a handheld myometer [1, 8]; however, the usage of more objective devices to test muscle strength, such as isokinetic dynamometers that enable robust assessments of the muscle strength of specific functional muscle groups, is still largely missing to evaluate the association between fat components and thigh muscle function. One previous study [22] focusing on the fat-strength interaction of quadriceps muscles by only testing a small cohort of healthy subjects used an isokinetic dynamometer for isometric strength measurement in knee extension and indicated that the quadriceps intermuscular adipose tissue fraction and intramuscular PDFF correlate significantly with physical strength, represented by the MVIC.

The present study confirms this first evidence of an interaction between muscular fat and strength by examining the associations between the PDFF and relMVIC at the thigh in healthy subjects and by showing not only significant correlations for the PDFF of the quadriceps muscles but also for ischiocrural muscles. Moreover, we were able to show that, in contrast to the relCSA, the PDFF of the quadriceps and ischiocrural muscles were significantly associated bilaterally with relMVIC in knee extension and flexion.

These new insights in the interactions of fat and strength parameters in human muscles demonstrate the importance of muscle quality, consisting of the individual number of muscular contractile elements, the specific fatty infiltration as well as the strength capacity [21]. The muscle quality might be able to predict and flesh out muscle (dys-)function a lot better than the relCSA does. Of note, there is no significant correlation between the relMVIC in flexion and the relCSA of the ischiocrural muscles for the left and right side. The anatomical difference (quadriceps muscles: one-joint muscles; ischiocrural muscles: multi-joint muscles) as well as the variations concerning the muscular structure and activation should be considered when interpreting this finding [35]. To specify the knowledge of muscle quality and (dys-)function in the future, further studies are needed to include and prove the meaning of the muscle volume and in particular the physiological CSA, the probably most powerful predictors concerning muscular strength [36–38].

Our observations seem to be in accordance with the finding of paraspinal PDFF being significantly correlated with the relMVIC in extension and flexion of the trunk, whereas paraspinal mean CSA only showed significant correlations with relMVIC in flexion in a previous study [39]. Furthermore, the suggested superiority of PDFF over relCSA for the prediction of muscle strength seems to complement previous work at the thigh region derived from patients with pathology, demonstrating negative correlations between the PDFF and muscle strength and indicating that muscle fat composition rather than muscle size correlates with knee extensor strength [5, 7–9, 40, 41].

It is important to now have evidence of correlations and predictions of PDFF and muscle strength also in healthy subjects as they clearly differ from patients with musculoskeletal disorders, metabolic diseases, or NMD regarding the ranges of PDFF measurements. PDFF values are generally much lower, and the produced strength values are higher in healthy subjects [1–9]. Knowledge about such correlations and our prediction model in muscles that are not or not yet pathologically fatty infiltrated allows the PDFF to become a biomarker and to potentially facilitate early treatment protocols or to arrange counteracting individual interventions such as changes in lifestyle or specific training programmes with individual adapted physical activities in order to maintain or improve muscle function. Former studies dealt with the clinical evaluation of the knee joint agonist-antagonist relationship [35, 37, 42–44]. Concerning knee dysfunctions and knee joint injuries, like ruptures of the anterior cruciate ligament, there is a high importance of the quadriceps-ischiocrural ratio. In our study, the connection of PDFF, relCSA, and relMVIC was as follows: in both genders, ischiocrural muscles were more infiltrated by fat than quadriceps muscles, had only about one third of the quadriceps relCSA, and produced about half of the quadriceps relMVIC. These insights pave the way for further research to prove the role of PDFF concerning muscle function and its role as an indicator to analyse and define muscle quality and fat-induced loss of muscle function as well as to develop corresponding training programmes dealing with these ratios. As a perspective, the role of physiological CSA, muscular pennation angle, and muscle volume could complement and specify muscle quality [32, 35].

Concerning methodology, chemical shift encoding-based water-fat MRI is confirmed as a fast method which can be added to routine MRI protocols of the thigh region to visualise and quantify muscle quality and possibly forecast deficits in muscle strength and function, leading over to subsequent specific training programmes. In this context, PDFF derived from chemical shift encoding-based water-fat MRI is a more robust and objective approach compared to the semi-quantitative, post-acquisitional analysis of conventional images [13]. However, the method requires compensation for confounding factors, reflected by T2* decay, a potential quantification bias due to multiple spectrum peaks, influence of eddy currents, and the T1 difference between fat and water compartments [28–30, 45]. Current sequences, such as the sequence used in this study, take these influencing factors into account. Regarding the muscle strength measurements, there is a need of familiarisation sessions prior to the testing visit to ensure to collect valid data. The rotational dynamometer testing procedure as well as the human ability to develop the real maximum of isometric strength are complex so that more visits are necessary to gain stable values of MVIC. Therefore, all participants performed at least one initial training session.

There are limitations to this study that we acknowledge. First, the comparatively small sample size. Future studies may enrol a larger sample size, which can be particularly realised by multicentre approaches using pre-existing and state-of-the-art imaging collected in joint databases [2]. Second, future studies may add magnetic resonance spectroscopy to explore the distribution of lipids within thigh muscles and distinctly quantify the intra- and extra-myocellular lipid levels [13]. Third, our approach of ROI placement was restricted to segmenting the ten most central slices whilst previous investigations used larger extension or semi-automated algorithms [2, 22, 46–48]. However, since we only included healthy subjects who were characterised by rather homogeneous fat distributions, a manual segmentation approach of only representative slices seems to be justified [22, 46]. Fourth, concerning the methodical way of strength measurement, there is a need of including physiological CSA, angle of pennation, and muscle volume in future studies as conclusive, modifying strength predictors and to integrate these values in prediction models [36, 49]. Fifth, the present study did not enrol patients suffering from musculoskeletal disorders, metabolic diseases, or NMD whilst insights in varying muscle quality of different top-performing athletes are still missing.

In conclusion, we observed correlations between the PDFF and relCSA and relMVIC at the thigh by quantitative MRI and precise measurements by a rotational dynamometer. In contrast to relCSA, the PDFF of the quadriceps and ischiocrural muscles was significantly associated bilaterally with the relMVIC in extension and flexion. Thus, chemical shift encoding-based water-fat MRI can provide important information and may potentially track early changes in muscles not pathologically fatty infiltrated. This might help to initiate early, individualised training protocols with specific physical activities in order to maintain or improve muscle function.

## Data Availability

The datasets used and analysed during the current study are available from the corresponding author on request.

## Abbreviations

BMI: Body mass index
CSA: Cross-sectional area
MRI: Magnetic resonance imaging
MVIC: Maximum voluntary isometric contraction
NMD: Neuromuscular diseases
PDFF: Proton density fat fraction
relCSA: Relative CSA
RelMVIC: Relative MVIC
ROI: Region of interest
TE: Echo time
TR: Repetition time
