# Correction to: Associations of thigh muscle fat infiltration with isometric strength measurements based on chemical shift encoding-based water-fat magnetic resonance imaging

**DOI:** 10.1186/s41747-019-0140-3

**Published:** 2019-12-23

**Authors:** Stephanie Inhuber, Nico Sollmann, Sarah Schlaeger, Michael Dieckmeyer, Egon Burian, Caroline Kohlmeyer, Dimitrios C. Karampinos, Jan S. Kirschke, Thomas Baum, Florian Kreuzpointner, Ansgar Schwirtz

**Affiliations:** 1 Department of Sport and Health Sciences, Technische Universität München, Georg-Brauchle-Ring 60/62, 80992 Munich, Germany; 2 Department of Diagnostic and Interventional Neuroradiology, Klinikum rechts der Isar, Technische Universität München, Ismaninger Str. 22, 81675 Munich, Germany; 3 Department of Diagnostic and Interventional Radiology, Klinikum rechts der Isar, Technische Universität München, Ismaninger Str. 22, 81675 Munich, Germany

**Correction to: Eur Radiol Exp (2019) 3:45**

**https://doi.org/10.1186/s41747-019-0123-4**

After publication of this article [1], it is noticed it contained some errors in the Methods section.

‘N*m^3^’ should be corrected to ‘N*m’ in the below three sentences and the correct version of the sentences are below:

1. Substantiated by measuring the isometric peak torque in Newton per metre (in N*m), the MVIC was produced in knee extension at 60° and knee flexion at 35°, which are the joint angles with the ideal muscular strength-length relationship to anticipate the real MVIC [31–34].
2. MVIC of each direction of movement (extension/flexion) was collected three times with 3 min of recovery in between, and the best value of muscle flexion and extension maximum isometric torque (in N*m) was respectively taken for data analysis [2, 22].
3. The measured absolute extension and flexion MVICs (in N*m) were adjusted for the individual BMI to obtain a relative MVIC (relMVIC, in N*m^3^/kg) for the left and right thigh muscles.

The original article has been corrected. We apologize for the inconvenience caused.
